# Cervical Spine Myelopathy Caused by Calcium Pyrophosphate Dihydrate Deposition of the Ligamentum Flavum: A Case Report

**DOI:** 10.7759/cureus.66869

**Published:** 2024-08-14

**Authors:** Henry Avetisian, Andy Ton, Thomas J Dowling, Raymond Hah

**Affiliations:** 1 Department of Orthopaedic Surgery, University of Southern California (USC) Keck School of Medicine, Los Angeles, USA; 2 Department of Orthopaedic Surgery, University at Buffalo Jacobs School of Medicine and Biomedical Sciences, Buffalo, USA

**Keywords:** cervical decompression, cervical radiculopathy, cervical myelopathy, calcium dyrophosphate deposition, pseudogout

## Abstract

Calcium pyrophosphate dihydrate deposition (CPPD), commonly known as pseudogout, is an inflammatory arthropathy primarily affecting the knee, wrist, hip, and shoulder joints. However, it can occasionally deposit in various structures surrounding the spinal column, including the facet joints, ligamentum flavum, bursae, and intervertebral discs. Such occurrences are typically asymptomatic or associated with mild neck pain. Nonetheless, severe cases may lead to myeloradiculopathy, characterized by severe neck pain and upper extremity weakness. Conservative management with nonsteroidal anti-inflammatory drugs is often sufficient for mild cases, while surgical decompression remains the gold standard for severe cases with significant spinal cord compression. Herein, we present a rare case of pseudogout, manifesting as cervical spine myelopathy due to calcium pyrophosphate dihydrate deposition in the ligamentum flavum and facet joints at C1-2. This was found incidentally during cervical spine decompression and fusion and subsequentially confirmed through pathological examination. Following the removal of the compressive pathology, the patient reported significant improvements in neck pain and neurological symptoms. This case underscores the importance of considering pseudogout in the differential diagnosis of acute neck pain presenting with myelopathy or radiculopathy.

## Introduction

Calcium pyrophosphate dihydrate deposition (CPPD), commonly referred to as pseudogout, is an inflammatory arthropathy characterized by the presence of CPPD in articular structures. Histologically, it is distinguished by the presence of weakly birefringent rhomboid crystals, contrasting with the negatively birefringent needle-shaped crystals above the gout [1]. The diagnosis of pseudogout poses challenges due to its varied clinical presentations, spanning from asymptomatic to severe inflammatory arthritis, often resembling rheumatoid arthritis and gout. Identified risk factors include osteoarthritis, advanced age, hemochromatosis, hypomagnesemia, hypophosphatasia, and hyperparathyroidism [2]. Typically, the calcium crystals associated with pseudogout are deposited in major weight-bearing joints such as the knees, hips, and shoulders.

In rarer instances, pseudogout can involve the spine, leading to the calcification of the ligamentum flavum in the cervical spine, ultimately resulting in spinal cord compression and myelopathy. The precise mechanism underlying this deposition remains unclear, although metabolic disorders, familial inheritance, and sporadic factors have been suggested [3].

Herein, we present a noteworthy case of myelopathy secondary to calcium pyrophosphate dihydrate deposition in the ligamentum flavum of the upper cervical spine, which was incidentally discovered during posterior cervical spinal decompression and fusion.

## Case presentation

A male patient in his late 70s with a past medical history of osteoarthritis, hypercholesterolemia, heart disease, depression, hypertension, and atrial fibrillation presented with a two-month history of worsening neck pain, accompanied by balance difficulties, dexterity issues, and right upper extremity weakness. 

A physical examination revealed restricted cervical spine mobility, tenderness in the cervical paraspinal muscles, and a positive Lhermitte sign. Additionally, there was evident weakness, hyperreflexia, and diminished sensation in the right upper limb, alongside bilateral lower extremity numbness and hyperreflexia, with a wide-based gait.

**Figure 1:**
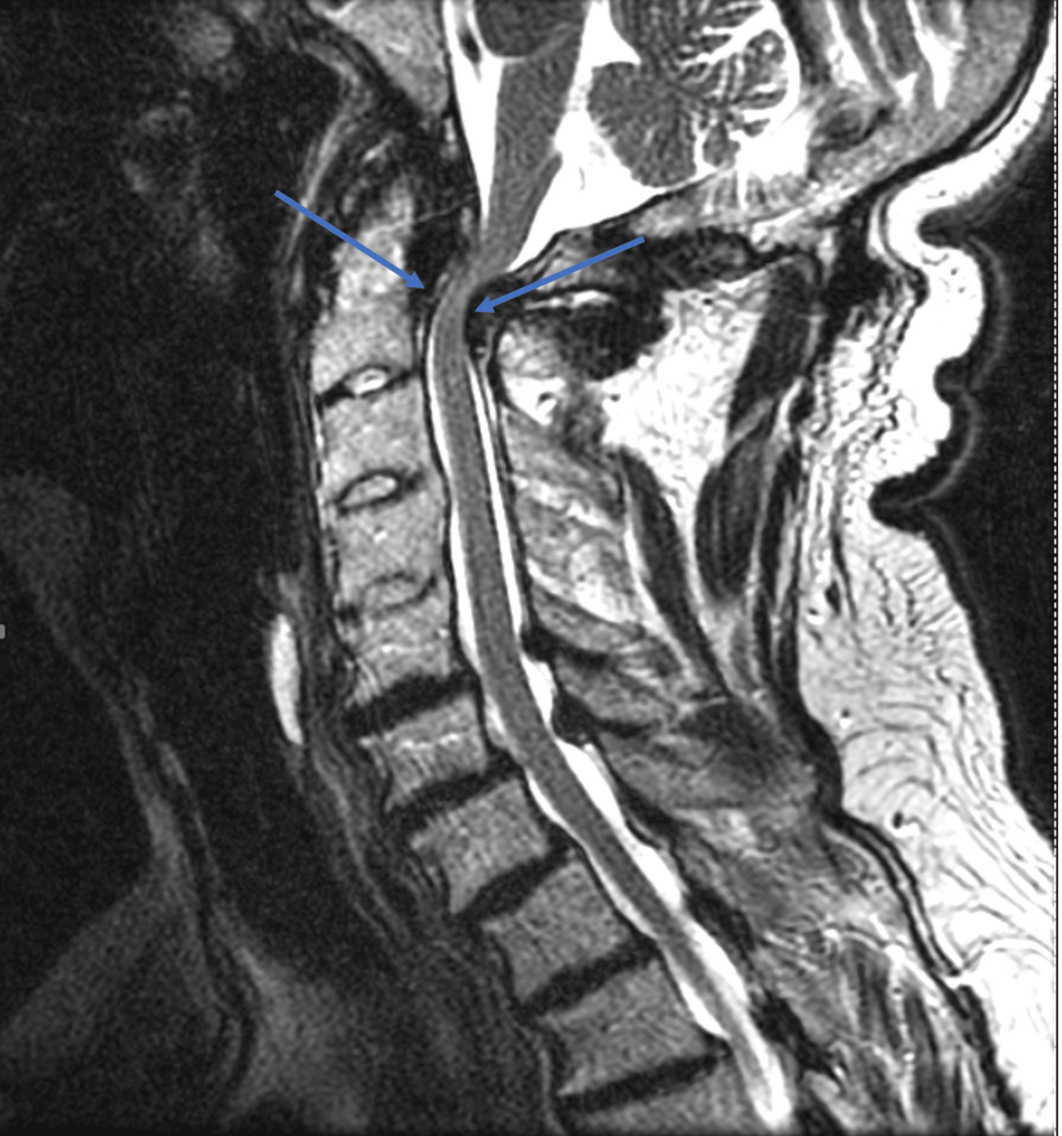
Cervical Spine MRI Sagittal T2-weighted MRI of the cervical spine. Arrows designate areas of severe stenosis at the C1-2 junction with both anterior and posterior compression.

**Figure 2:**
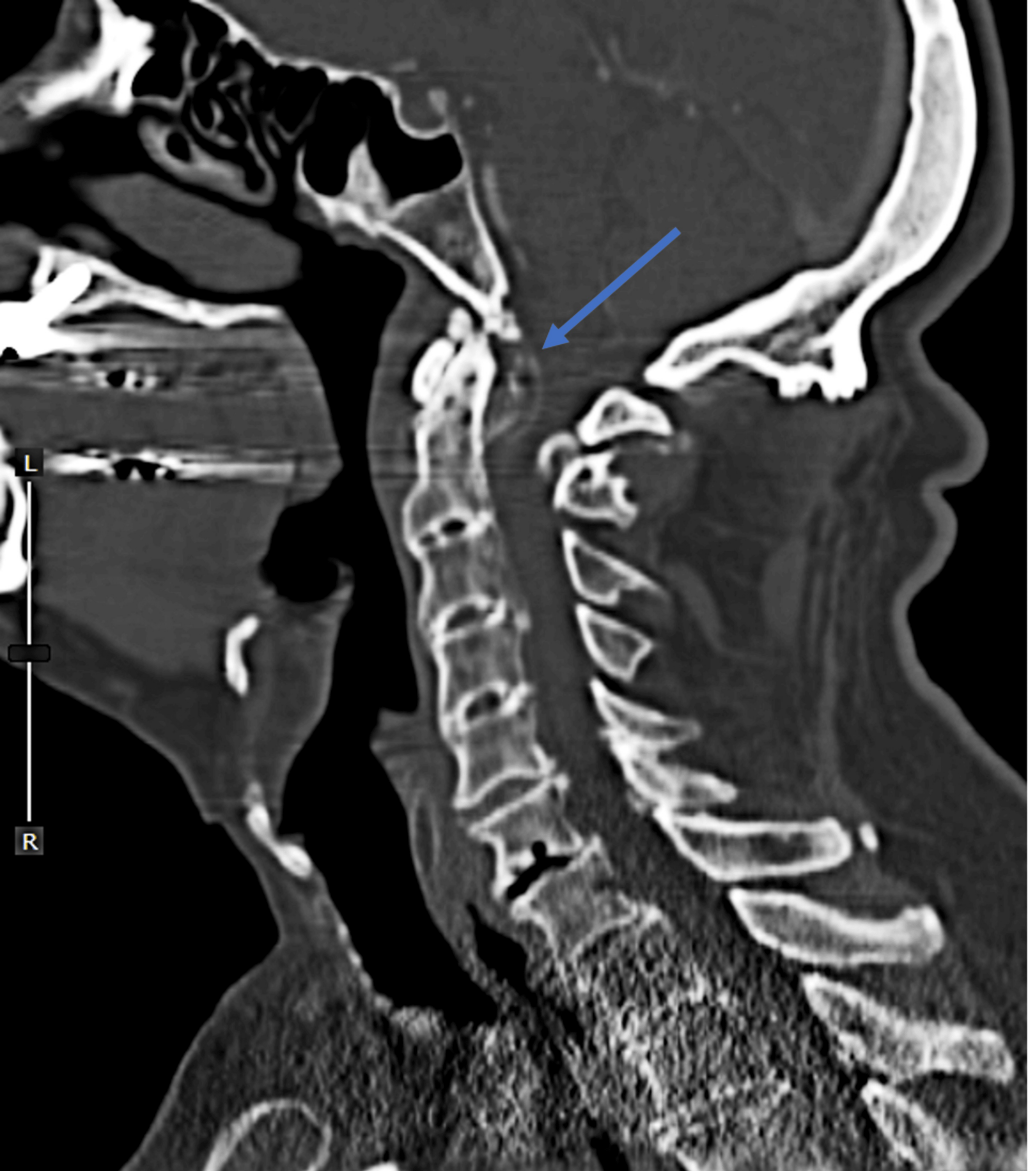
Cervical Spine CTA Midsagittal plane CTA of the cervical spine demonstrating a retrodental calcified compressive mass (blue arrow).

Magnetic resonance imaging (MRI) of the cervical spine (Figure 1) revealed significant stenosis at the C1-C2 junction due to anterior and posterior compression, alongside fusion spanning from C3 to C5, with indications of myelomalacia. Cervical spine CTA (Figure 2) displayed retro-dental compressive mass and inflammatory changes.

Clinical presentation, examination findings, and imaging results were consistent with degenerative cervical myelopathy. Given the patient's symptomatic progression and failure of conservative management, including epidural steroid injections and physical therapy, a decision was made to proceed with a C1-2 laminectomy and a C1-C3 posterior spinal fusion to allow better visualization of the posterior spinal column.

During the surgical intervention, notable crystalline deposition was observed within the ligamentum flavum at C1-C2, along with the involvement of the facet joints, raising suspicion of pseudogout. Tissue samples were obtained for pathological examination, confirming calcium pyrophosphate dihydrate deposition. Excision of the pathological ligamentum flavum and decompression were successfully completed without complications. 

At the six-week follow-up, the patient reported complete resolution of right upper extremity radiculopathy and a notable improvement in gait stability. He no longer relied on a walker and only occasionally utilized a cane for ambulation. Additionally, improved manual dexterity enabled him to perform tasks such as buttoning his shirts with ease.

## Discussion

This patient case underscores a rare presentation of pseudogout, which is typically associated with larger weight-bearing joints and bursae, affecting the ligamentum flavum and facet joints of the cervical spine. As spine surgeons, it is vital to note that these crystals can deposit in the ligamentum flavum, facet joints, and other structures that are near the spinal cord and that their presence can severely hinder neurological function. This highlights the need to keep inflammatory arthropathies such as pseudogout in the differential diagnosis when a patient presents with acute neck pain and cervical myelopathy versus radiculopathy.

While this patient exhibited neck pain, stiffness, and upper extremity weakness, the clinical spectrum of CPPD is diverse. In some patients, neck pain may radiate to the shoulder and occipital region, mimicking polymyalgia rheumatica [4]. Acute CPPD attacks may also present with nonspecific symptoms such as headache and fever, mimicking meningitis [4].

Histological confirmation of pseudogout in our case, via pathological examination of the ligamentum flavum, was crucial for diagnosis. However, imaging before the surgery did not initially reveal findings suspicious for CPPD. Kobayashi et al. [5] reported a similar case of CPPD in the ligamentum flavum of the cervical spine. Preoperatively, on a CT scan, they discovered a high-density area between the C5 and C6, raising suspicion of pseudogout. MRI revealed intermediate-signal intensity on T1WI and a high signal intensity on T2WI surrounding a low signal region on both T1WI and T2WI with spinal cord compression, suggesting pseudogout attack [5]. Their patient also demonstrated some of the clinical characteristics of inflammatory arthritis, such as fever, an elevated white blood cell count, and C-reactive protein. Furthermore, other radiologic manifestations of CPPD in the cervical spine include chondrocalcinosis, which can be seen on X-rays, MRIs, and CT scans [6]. However, this is a nonspecific finding and can also be seen in amyloidosis and hyperparathyroidism [6]. Thus, the only way to confirm pseudogout is through histologic examination. However, MRI remains the optimal imaging modality for detecting pseudogout due to its superior soft tissue contrast [5].

In our patient's case, we were able to treat the compressive pathology caused by the pseudogout through surgical decompression. However, the management of the disease can vary by patient, depending on the course of the disease. In patients who present with only mild spinal cord compression and with elevated C-reactive protein, conservative treatments such as NSAIDs, corticosteroids, and low-dose colchicine are effective [3]. Surgical decompression through laminectomy remains the gold standard treatment in patients with severe myelopathy and negative inflammatory markers. A reported complication of surgical removal of CPPD is dural tears, as these crystals can tightly adhere to the dura mater [3]. Furthermore, in cases of prolonged CPPD, the spinal cord damage can be irreversible, so neurological symptoms will persist even after the removal of the crystal deposits.

There are limitations in this case report. First, the patient had multiple pathologies in the cervical spine; thus, it is difficult to determine which of the patient's symptoms can be attributed to CPPD. Second, due to the short follow-up, we are not able to determine at this time if his CPPD will recur. 

## Conclusions

Calcium pyrophosphate dihydrate deposition (CPPD) can occur in the ligamentum flavum and posterior elements of the cervical spine, contributing to cervical stenosis, cord compression, and myelopathy, which presents as neck pain and cervical radiculopathy. A biopsy is required to confirm the diagnosis; however, preoperative MRI can detect calcification. Although only required in severe cases, decompressive surgery is associated with a good prognosis.
